# OsRAD51C is essential for double-strand break repair in rice meiosis

**DOI:** 10.3389/fpls.2014.00167

**Published:** 2014-05-07

**Authors:** Ding Tang, Chunbo Miao, Yafei Li, Hongjun Wang, Xiaofei Liu, Hengxiu Yu, Zhukuan Cheng

**Affiliations:** ^1^State Key Laboratory of Plant Genomics and Center for Plant Gene Research, Institute of Genetics and Developmental Biology, Chinese Academy of SciencesBeijing, China; ^2^Key Laboratory of Plant Functional Genomics of Ministry of Education, Yangzhou UniversityYangzhou, China

**Keywords:** rice, meiosis, OsRAD51C, double-strand break repair, chromosome fragmentation

## Abstract

RAD51C is one of the RAD51 paralogs that plays an important role in DNA double-strand break repair by homologous recombination. Here, we identified and characterized OsRAD51C, the rice homolog of human RAD51C. The *Osrad51c* mutant plant is normal in vegetative growth but exhibits complete male and female sterility. Cytological investigation revealed that homologous pairing and synapsis were severely disrupted. Massive chromosome fragmentation occurred during metaphase I in *Osrad51c* meiocytes, and was fully suppressed by the *CRC1* mutation. Immunofluorescence analysis showed that OsRAD51C localized onto the chromosomes from leptotene to early pachytene during prophase I, and that normal loading of OsRAD51C was dependent on OsREC8, PAIR2, and PAIR3. Additionally, ZEP1 did not localize properly in *Osrad51c*, indicating that OsRAD51C is required for synaptonemal complex assembly. Our study also provided evidence in support of a functional divergence in RAD51C among organisms.

## Introduction

The maintenance of genome stability is essential for cell and organism viability. Both environmental and endogenous DNA damaging agents, including ionizing radiation, chemicals, and spontaneous DNA breakage during DNA recombination and replication can cause DNA lesions (Ward, 1988; Friedberg et al., 1995; Olive, 1998; Flores-Rozas and Kolodner, 2000). Among these lesions, DNA double-strand breaks (DSBs) are one of the most disruptive forms of DNA damage (Jackson and Bartek, 2009). Unrepaired DSBs can lead to severe chromosomal aberrations that may ultimately result in apoptosis or aneuploidy. In response to the threats posed by DNA damage, organisms have evolved effective mechanisms for DSB repair, which include two major pathways: homologous recombination (HR) and non-homologous end joining (NHEJ). HR utilizes the sister chromatid as a template for new DNA synthesis, guaranteeing the delivery of stable genetic information to the next generation (Van Den Bosch et al., 2002; Sonoda et al., 2006). In contrast, NHEJ completes DSB repair by directly ligating the DNA break ends without using a homologous template. Therefore, due to this imprecise repair mechanism, inappropriate NHEJ is potentially mutagenic and can lead to chromosome anomalies (Lees-Miller and Meek, 2003; Lieber, 2008).

Meiotic recombination is initiated by SPO11-mediated DSBs. In *Saccharomyces cerevisiae*, Spo11 plays a major role in catalyzing the formation of DSBs via its topoisomerase-like transesterase activity (Keeney et al., 1997; Keeney, 2001). Recent studies indicate that the role of SPO11 in the initiation of meiotic recombination seems to be highly conserved, because homologs of the *SPO11* gene have been found in a wide range of organisms (Dernburg et al., 1998; McKim and Hayashi-Hagihara, 1998; Hartung and Puchta, 2000; Romanienko and Camerini-Otero, 2000; Yu et al., 2010). Numerous genes involved in HR have been identified recently in various organisms. Rad51, a eukaryotic homolog of bacterial RecA protein, has DNA-dependent ATPase activity and facilitates strand exchange between homologous DNA molecules (Baumann and West, 1998). Rad51 is conserved from yeast to human in both structure and function (Bezzubova et al., 1993; Shinohara et al., 1993). In addition, five RAD51 paralog proteins (RAD51B, RAD51C, RAD51D, XRCC2, and XRCC3) have also been studied in mammalian cells and are thought to play important roles in the process of recombination repair (Albala et al., 1997; Dosanjh et al., 1998; Liu et al., 1998; Pittman et al., 1998; Johnson et al., 1999; Pierce et al., 1999; Bleuyard et al., 2005). These proteins have 20–30% similarity and possess common functional domains (Miller et al., 2004). Biochemical and yeast two-hybrid analyses indicate that RAD51 paralogs form two distinct protein complexes: a RAD51B-RAD51C-RAD51D-XRCC2 (BCDX2) complex and a RAD51C-XRCC3 (CX3) complex (Masson et al., 2001; Miller et al., 2002; Wiese et al., 2002; Yokoyama et al., 2004). Consistent with this finding, a RAD51C-defective hamster cell line fails to form either of these two complexes, implying that RAD51C plays a prominent role in the HR processes (French et al., 2002).

The *RAD51C* gene was originally identified in humans as a new member of the RAD51 family by sequence similarity (Dosanjh et al., 1998). The first mammalian RAD51C-defective mutant was the RAD51C-deficient Chinese hamster cell line CL-V4B. CL-V4B displayed increasing numbers of spontaneous chromosomal aberrations and was highly hypersensitive to cross-linking agents such as mitomycin C (MMC) and cisplatin (Godthelp et al., 2002; Drexler et al., 2004). Subsequently, similar phenotypes were observed in both *RAD51C*-depleted chicken DT cells and HeLa cells (Takata et al., 2001; Lio et al., 2004). In addition, a RAD51C-like protein, Spn-D, has been identified in Drosophila, and shows 38% identity with human RAD51C. Intriguingly the *spn-D* mutant is insensitive to X-rays and methyl methanesulfonate, suggesting that Spn-D may play a role only in meiotic recombination rather than DNA damage repair in somatic cells (Abdu et al., 2003). In contrast, RAD51C is essential for early development in mammals, since deficiency leads to embryonic lethality in the mouse (Kuznetsov et al., 2009). With the help of the hypomorphic allele of the *rad51c* mutant, further study revealed that the *rad51c* mutation results in early prophase I arrest in males and broken chromosomes or aneuploidy at metaphase II in female mice (Kuznetsov et al., 2007). In the higher plant, Arabidopsis, the *atrad51c* mutant displayed complete sterility during flowering. In addition, it was sensitive to γ-radiation and cisplatin during vegetative development and demonstrated reduced HR frequencies and increased chromosome fragments in somatic cells (Abe et al., 2005; Li et al., 2005).

An increasing number of genetic and biochemical studies have provided further understanding of the precise role of RAD51C in HR. RAD51C exhibits an ATP-independent DNA strand exchange activity, DNA-stimulated ATPase activity, and ssDNA binding activity *in vitro* (Lio et al., 2003), suggesting it may participate in both early and late HR events. Consistent with this, it has been reported that RAD51C not only promotes RAD51 assembly in the early stages of recombination (French et al., 2002), but is also involved in branch migration and Holliday junction (HJ) resolution in mammalian cells (Liu et al., 2004). However, RAD51C does not directly interact with the human Holliday junction resolvase GEN1, which binds specifically to and promotes resolution of HJs (Ip et al., 2008; Rass et al., 2010). The role of RAD51C in the late stages of HR remains obscure. Furthermore, RAD51C can also facilitate CHK2 phosphorylation after irradiation and thereby promotes the transduction of damage signals (Badie et al., 2009).

A RAD51C homolog has recently been characterized in rice. It has been reported that OsRAD51C is required for both male and female gamete development, and its mutant exhibits sensitivity to DNA damaging agents (Kou et al., 2012). However, the specific functions of OsRAD51C in meiotic recombination, its spatial and temporal expression pattern, and its relationships with other determinant proteins in meiosis remain obscure. In this study, we identified another *Osrad51c* mutant showing a similar phenotype to that reported by Kou et al. By studying chromosome behavior in *Osrad51c*, we found that OsRAD51C is required for processing of DSBs, homologous pairing, and synaptonemal complex (SC) assembly. Furthermore, OsRAD51C shows a localization pattern in rice that is distinct from that of its homolog in mice. Our results therefore provide further clues to the role of OsRAD51C in meiosis.

## Materials and methods

### Plant materials

The rice (*Oryza sativa* L.) *Osrad51c* mutant, induced by ^60^Co γ-ray radiation, was isolated from the *indica* rice variety Zhongxian 3037. We then generated the *Osrad51c crc1* double mutant by crossing heterozygous *Osrad51c*± and *crc1*± and selecting double mutants in the F2 progeny. The meiotic mutants used in this study, including *Osrec8*, *pair2*, *pair3*, and *crc1*, have been reported in previous work (Shao et al., 2011; Wang et al., 2011; Miao et al., 2013). All plants were cultivated in paddy fields under normal growth conditions.

### Map-based cloning of *OsRAD51C*

The F2 mapping populations were constructed from a cross between heterozygous *Osrad51c*± and a *japonica* cultivar, Zhonghua11, and 898 sterile plants were selected for isolating the target gene. Sequence-tagged site (STS) markers were developed based on sequence differences between the *indica* variety 9311 and the *japonica* variety Nipponbare according to the data published on the NCBI website (http://www.ncbi.nlm.nih.gov). All primer sequences are listed in Supplemental Table 1.

### RNAi analysis

For the RNAi construct, a 470-bp fragment of the *OsRAD51C* cDNA sequence was amplified using the primer pairs CR1 (Supplemental Table 1). RNAi vector construction and transformation were performed as previously described (Wang et al., 2009).

### Antibody production

To generate antibody against OsRAD51C, a 210-bp fragment of *OsRAD51C* cDNA was amplified using primer CA1 (Supplemental Table 1). Vector construction, fusion peptide expression, and protein purification were performed as previously described (Wang et al., 2009). The fusion peptide was used to immunize mice to generate anti-OsRAD51C polyclonal antibody. The polyclonal antibodies to OsREC8, PAIR2, PAIR3, and ZEP1 have been described previously (Wang et al., 2010; Shao et al., 2011; Wang et al., 2012).

### Meiotic chromosome preparation and cytological analysis

Young panicles were harvested and fixed in Carnoy's fixative solution and stored at −20°C. Microsporocytes at the appropriate meiotic stage were squashed and stained with acetocarmine. After washing with 45% acetic acid, the chromosome preparations were frozen in liquid nitrogen. After removing the coverslips, the slides were dehydrated through an ethanol series (70, 90, and 100%). Chromosomes were counterstained with 4,6-diamidinophenylindole (DAPI) in an antifade solution (Vector Laboratories, Burlingame, CA, USA). Images were captured under the ZEISS A2 fluorescence microscope with a micro CCD camera.

### Immunofluorescence assays

Fresh young panicles were fixed in 4% (w/v) paraformaldehyde for 10–30 min at room temperature. Anthers were squashed on a slide in 1× PBS solution. After freezing in liquid nitrogen and quickly removing the coverslip, the slides were dehydrated through an ethanol series (70, 90, and 100%). The slides were then incubated in a humid chamber at 37°C for 4 h in different combinations of primary antibodies diluted 1:500 in TNB buffer (0.1M Tris-HCl, pH 7.5, 0.15M NaCl, and 0.5% blocking reagent). After three washes in 1× PBS, slides were incubated with the Texas-red-conjugated goat anti-rabbit antibody and fluorescein isothiocyanate-conjugated sheep anti-mouse antibody at 37°C for 2 h, and again washed three times in 1× PBS. Finally, the slides were counterstained with DAPI in an antifade solution. Images were captured by a fluorescence microscope.

## Results

### Morphological characterization of the *Osrad51c* mutant

A sterile mutant was isolated from the progeny of Zhongxian 3037 treated with γ-ray radiation, and named *Osrad51c*. The mutant plant was normal during vegetative growth, but exhibited complete spikelet sterility. To further investigate the fertility of the male gametes, we examined mature pollen viability in *Osrad51c* by staining with iodine potassium iodide solution (I_2_-KI). Only empty, shrunken and unshaped pollens were observed, indicating that microspores were inviable. When pollinated with wild-type pollen, the mutant plants still did not produce any seeds, demonstrating that megaspore development was also affected. These results suggest that the sterile phenotype in *Osrad51c* was caused by both male and female sterility.

### Isolation and identification of the *OsRAD51C* gene in rice

To identify the mutated gene, a map-based cloning approach was carried out in an F2 population, which was constructed by crossing the heterozygous *Osrad51c*± to a *japonica* cultivar Zhonghua11. The *OsRAD51C* gene was initially mapped to the long arm of chromosome 1 and further narrowed to a 19-kb region. Within this region, one candidate gene (Os01g0578000) was identified and annotated as a DNA repair and recombination protein. Phylogenetic analysis revealed that it is a rice homolog of human *RAD51C* that has been previously reported by Kou et al. (2012). Sequencing revealed an A-to-T single base mutation within the ninth exon of the *OsRAD51C* gene, which leads to premature protein termination. To further confirm that *OsRAD51C* was the gene responsible for the observed phenotype, we generated transgenic rice plants in which *OsRAD51C* transcription was downregulated using the RNAi approach. As predicted, the transgenic plants exhibited complete pollen and spikelet sterility, providing confirmation that the phenotype of *Osrad51c* is caused by mutation of the *OsRAD51C* gene.

### Chromosome behaviors in the *Osrad51c* mutant

**Figure 1:**
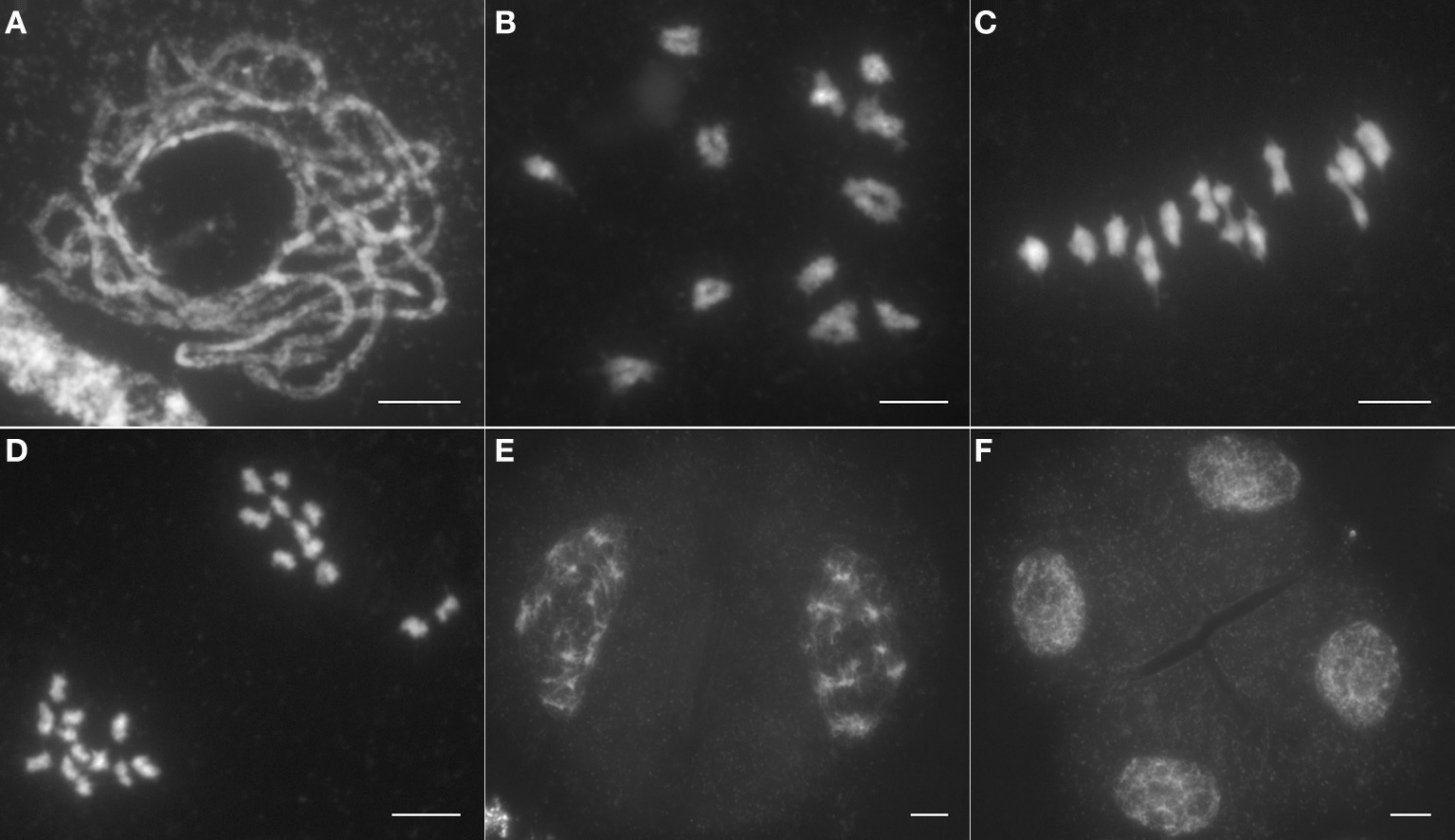
**Male meiosis in wild type. (A)** Pachytene; **(B)** Diakinesis; **(C)** Metaphase I; **(D)** Anaphase I; **(E)** Dyad; **(F)** Tetrad. Bars, 5 μm.

To ascertain the causes of the sterile phenotype in *Osrad51c*, we investigated meiotic progression in pollen mother cells (PMCs) from both wild-type plants and the *Osrad51c* mutant. In the wild-type, representative meiotic stages were observed: chromosomes began to condense and appeared as long thin threads at leptotene; pairing and initial synapsis occurred between homologous chromosomes at the zygotene stage; homologs became tightly associated and fully synapsed during pachytene (Figure 1A). After further chromosome condensation at diplotene, 12 short bivalents were clearly observed at diakinesis (Figure 1B), and these aligned on the equatorial plate during metaphase I (Figure 1C). From anaphase I to telophase I, homologous chromosomes separated from each other and migrated to opposite poles (Figure 1D). In meiosis II, sister chromatids separated and tetrads were generated (Figures 1E,F).

**Figure 2:**
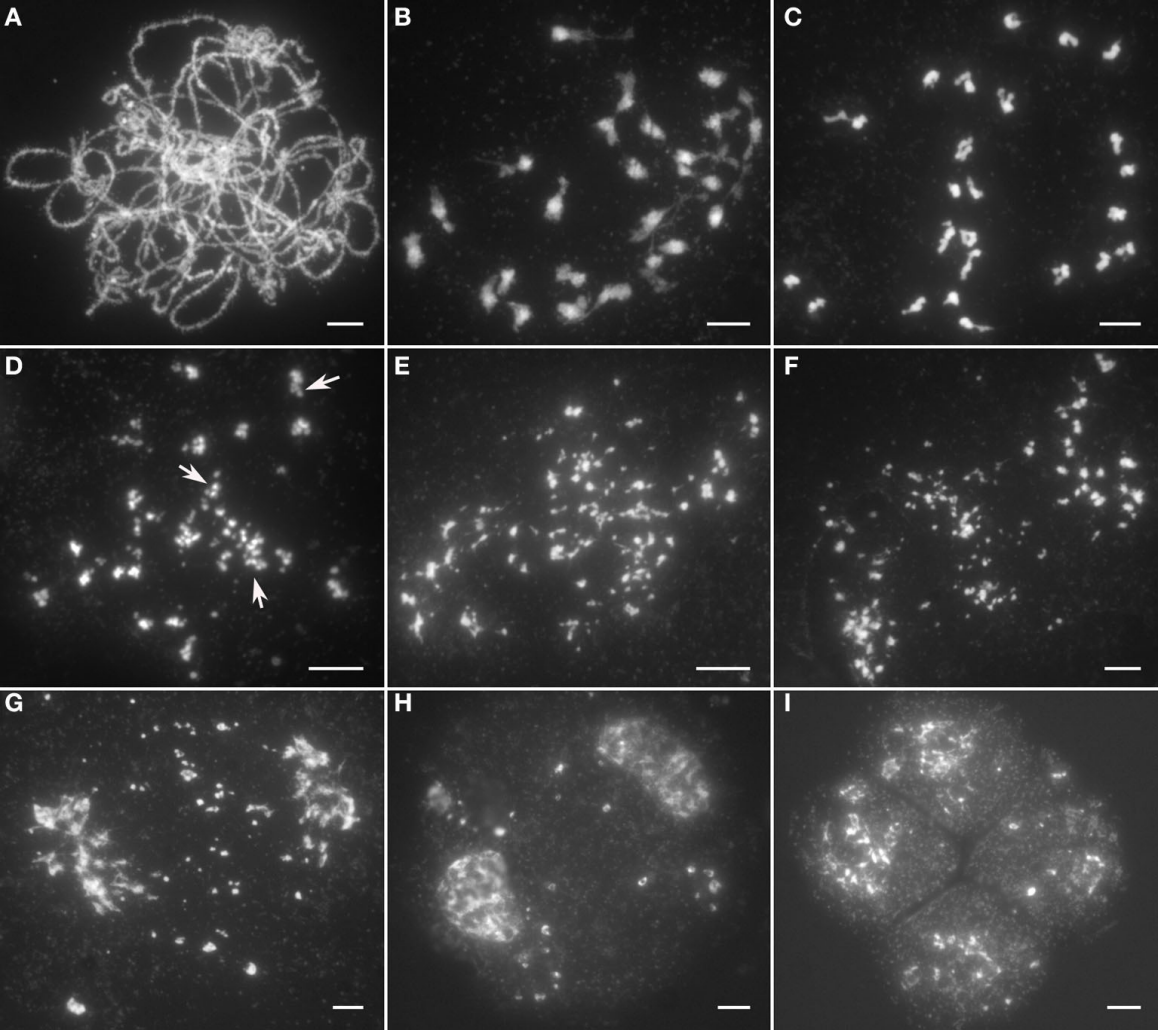
**Male meiosis in the *Osrad51c* mutant. (A)** Pachytene; **(B)** Diakinesis; **(C)** Early metaphase I; **(D)** Middle metaphase I; **(E)** Late metaphase I; **(F)** Anaphase I; **(G)** Telophase I; **(H)** Dyad; **(I)** Tetrad. Bars, 5 μ m.

Chromosomal behavior at leptotene in *Osrad51c* was quite similar to the wild-type. However, obvious defects in meiotic chromosomes were first observed at zygotene, when *Osrad51c* chromosomes remained as single threads and did not pair up with their homologs. Fully synapsed homologs were never observed at the pachytene stage in *Osrad51c* mutants (Figure 2A). At the diakinesis stage, following further condensation, 24 irregularly shaped univalents were observed in the PMCs (Figure 2B). At early metaphase I, the univalents did not align along the equatorial plate and remained scattered throughout the nucleus (Figure 2C). Meanwhile, a few small pieces which had broken away from the main chromosome were detected for some univalents (Figure 2D, arrows). Subsequently, the number of chromosomal fragments increased markedly in a short time, and became randomly distributed throughout the entire nucleus (Figure 2E). Despite the massive genome fragmentation, the meiotic cell cycle still entered into anaphase I, with the chromosomes asynchronously migrating toward the two opposite poles and acentric chromosomal fragments becoming randomly scattered in the nucleus (Figure 2F). At telophase I, when the major chromosomes arriving at the poles started to decondense, many lagging fragments could still be seen excluded from the nuclei (Figure 2G). They finally led to the formation of numerous micronuclei in the subsequent dyads and tetrads (Figures 2H,I). We therefore concluded that the sterility of the *Osrad51c* mutant is caused by defective homologous chromosome pairing and massive chromosomal fragmentation. Similar abnormalities were also observed in *OsRAD51C* RNAi plant meiocytes.

### DSB formation is a prerequisite for chromosome fragmentation in *Osrad51c*

**Figure 3:**
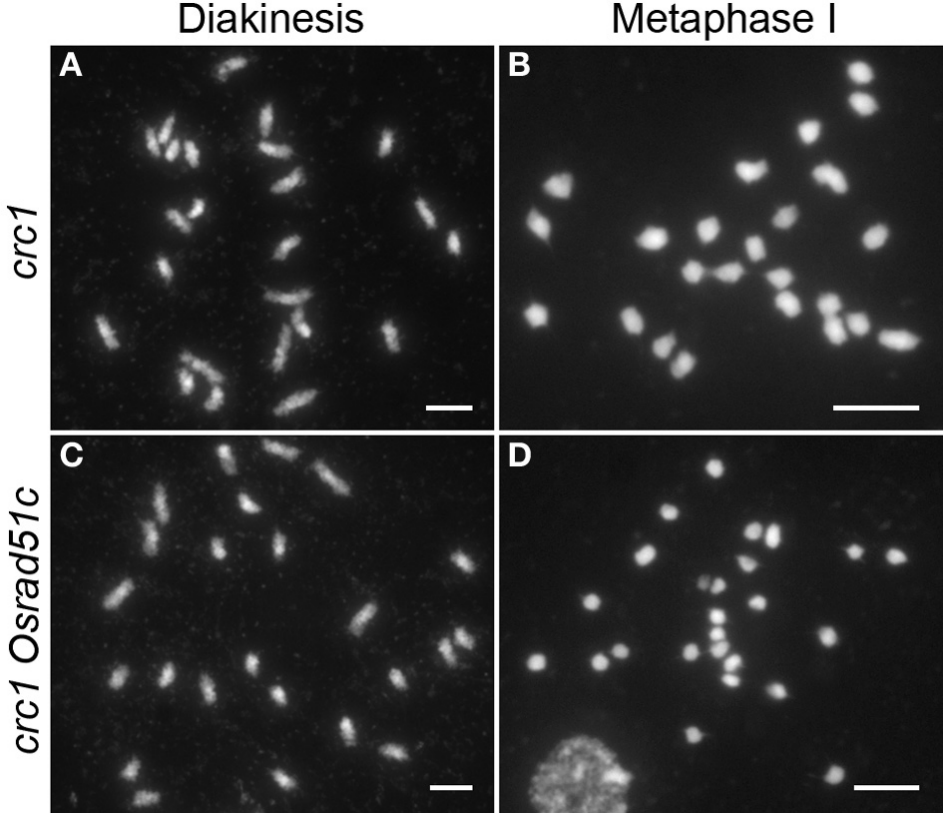
**Male meiosis in *crc1* (A,B) and *crc1 Osrad51c* (C,D). (A,C)** Diakinesis; **(B,D)** Metaphase I. Bars, 5 μm.

To further clarify whether chromosome fragmentation resulted from unrepaired DSBs, we generated *Osrad51c crc1* double mutants. CRC1, a component of the central region of the SC in rice, is required for meiotic chromosome pairing and recombination, and DSB formation is completely abolished in the absence of CRC1 (Miao et al., 2013). In the *crc1* single mutant, homologous chromosome pairing and synapsis were defective and 24 univalents were seen at diakinesis and metaphase I (Figures 3A,B). However, in the *Osrad51c crc1* double mutant, chromosomal behavior was very similar to that in *crc1*. No DNA fragments were detected; instead, only 24 intact univalents were observed at both diakinesis and metaphase I in *Osrad51c crc1* (Figures 3C,D). The massive chromosomal fragmentation seen in *Osrad51c* was completely suppressed by the defective CRC1. We therefore concluded that failure of meiotic DSB repair is responsible for the massive chromosomal fragmentation in *Osrad51c*.

### OsRAD51C localizes to meiotic chromosomes from leptotene to early pachytene

**Figure 4:**
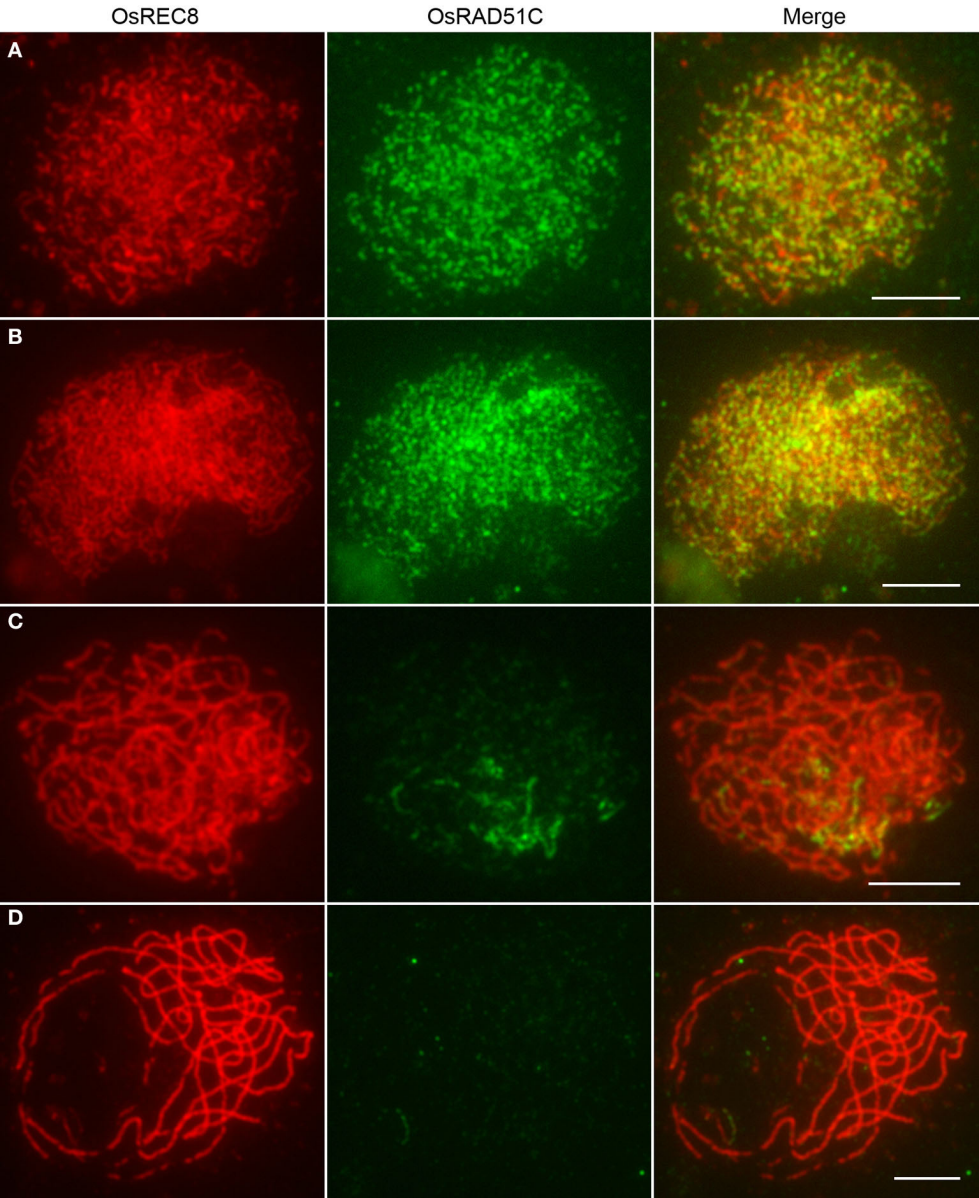
**Dual immunolocalization of OsREC8 (red) and OsRAD51C (green) in wild type PMCs. (A)** Leptotene; **(B)** Zygotene; **(C)** Mid prophase I; **(D)** Late pachytene. Bars, 5 μm.

In order to investigate the spatial and temporal localization patterns of OsRAD51C protein during meiosis, immunofluorescence assays were performed using polyclonal antibodies against OsRAD51C and OsREC8, from mice and rabbits, respectively. REC8, a key component of the sister chromatid cohesion complex, is required for axial element formation and homolog pairing. OsREC8 is first detectable as a diffuse signal during premeiotic interphase and gradually elongates along the entire length of the chromosomes from leptotene to pachytene. The signal eventually disappears at metaphase I. Consequently, OsREC8 has frequently been used as a chromosome marker in prophase I (Shao et al., 2011). The OsRAD51C signals were first detected as punctuate foci at leptotene (Figure 4A). The number of these foci gradually increased and reached a maximal level at zygotene (Figure 4B). Subsequently, OsRAD51C protein began to rapidly dissociate from the chromosomes. Once meiosis entered early pachytene, only a few residual signals could be seen (Figure 4C). At late pachytene, the OsRAD51C had completely disappeared from the chromosomes and was not detected in subsequent stages (Figure 4D). We therefore conclude that OsRAD51C plays a role in homologous chromosome pairing and DSB repair from leptotene to early pachytene in rice meiosis.

### OsRAD51C is required for normal SC assembly

**Figure 5:**
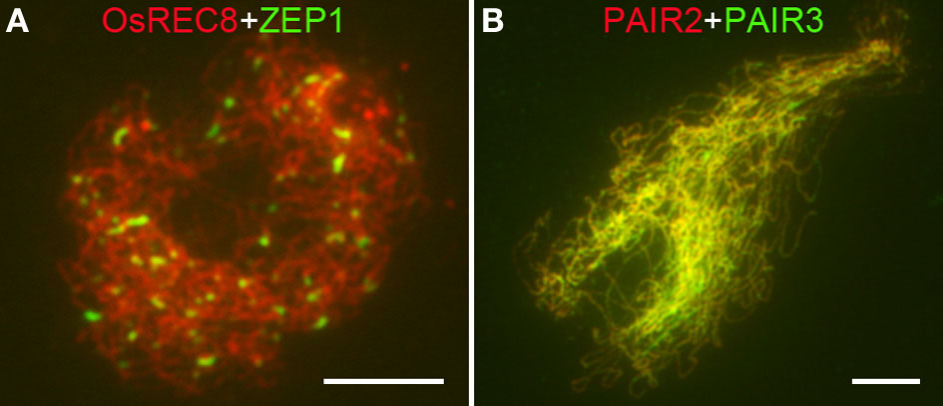
**Dual immunolocalization of several meiotic elements in *Osrad51c* PMCs. (A)** OsREC8 (red) and ZEP1 (green) signals at early pachytene; **(B)** PAIR2 (red) and PAIR3 (green) signals at early pachytene. Bars, 5 μ m.

The formation of SCs accompanies meiotic recombination and thus serves as a cytological marker of mature HR. To determine whether SC formation was affected in *Osrad51c*, we further investigated the localization of ZEP1, PAIR2, and PAIR3. PAIR2, the homolog of *S. cerevisiae* HOP1 and *Arabidopsis* ASY1, which associates with the axial element/lateral element (AE/LE), is required for homologous chromosome synapsis during early prophase I in rice (Nonomura et al., 2006). ASY1 foci also serve as a marker to detect chromosome axis formation both in *Arabidopsis* and barley (Chelysheva et al., 2005; Wang et al., 2010; Higgins et al., 2012). PAIR3 is an AE/LE associated protein, which is essential for homologous pairing, normal recombination, and SC assembly (Wang et al., 2011). ZEP1, a transverse filament protein, is the central element of the SC (Wang et al., 2010). These three proteins were used to monitor the assembly of SCs in *Osrad51c* meiocytes. We found that PAIR2 and PAIR3 in *Osrad51c* behaved in the same manner as in the wild-type. Both of them were co-localized and distributed along the chromosomes (Figure 5B), indicating that the axial elements of SCs were able to assemble completely in the absence of OsRAD51C. In the wild-type prophase I, ZEP1 signals were first visible as punctuate foci at leptotene, then quickly elongated to form linear signals along the entire chromosomes at early pachytene. However, in *Osrad51c* meiocytes, SC extension was blocked, and only short discontinuous linear signals were observed at pachytene (Figure 5A), suggesting the failure of SC assembly in *Osrad51c*. Together, although the formation of AEs appeared normal, the central element of the SC was not completely assembled, demonstrating that mature SC formation was disrupted in the absence of OsRAD51C.

### OsRAD51C localization relies on *OsREC8*, *PAIR2*, and *PAIR3*, but not on *ZEP1*

**Figure 6:**
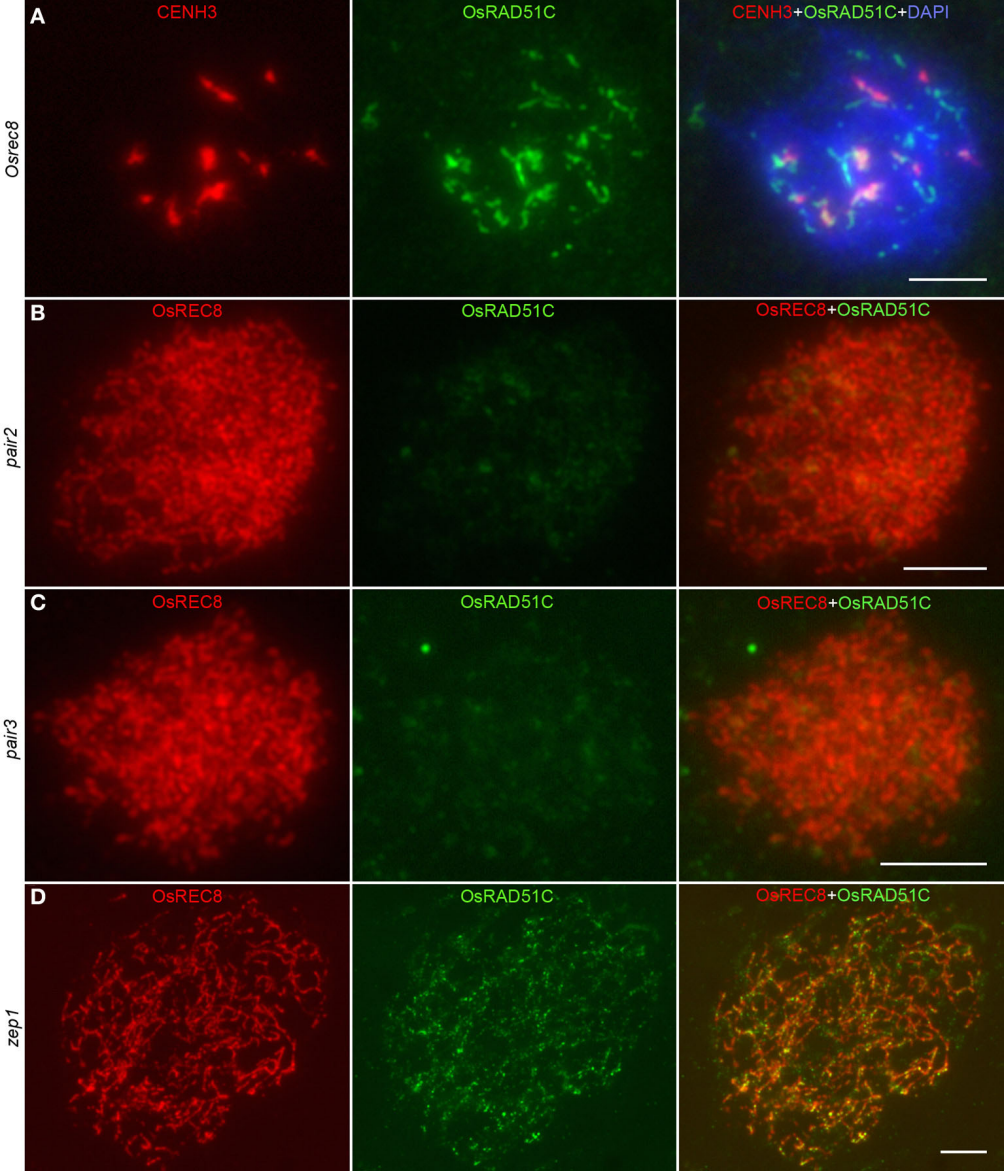
**The localization of OsRAD51C at leptotene in different meiotic mutants of rice. (A)** *Osrec8*; **(B)** *pair2*; **(C)** *pair3*; **(D)** *zep1*. The CENH3 signals (red) indicating the PMC in *Osrec8*, and the OsREC8 signals (red) indicating the PMCs in *pair2*, *pair3*, and *zep1*. OsRAD51C signals in green. Chromosomes stained with DAPI (blue). Bars, 5μ m.

To further examine OsRAD51C function in rice meiosis, we investigated the recruitment of OsRAD51C in *Osrec8, pair2, pair3, and zep1* mutants by immunofluorescence. In *Osrec8*, we employed CENH3 as a centromere marker to indicate the PMCs. Unlike the discrete dot-like signals at zygotene in the wild-type, OsRAD51C signals were gathered into bigger and brighter aggregates in *Osrec8* (Figure 6A), implying that correct loading of OsRAD51C requires OsREC8. We then examined the location of OsRAD51C proteins in the other three mutants using anti-OsREC8 antibody as a chromosome marker. We failed to detect any OsRAD51C signals in both the *pair2* and *pair3* mutants (Figures 6B,C), suggesting that OsRAD51C loading also relies on PAIR2 and PAIR3. However, fine punctuate foci of OsRAD51C protein were distributed normally along the chromosomes in *zep1* zygotene meiocytes (Figure 6D), indicating that defective ZEP1 does not affect OsRAD51C loading. We therefore concluded that normal localization of OsRAD51C depends on OsREC8, PAIR2, and PAIR3, rather than ZEP1.

## Discussion

### The role of OsRAD51C in rice meiosis

Meiotic recombination is initiated by the deliberate introduction of DSBs. Repair of these SPO11-mediated DSBs is a complicated process, involving multiple proteins via various pathways. RAD51C is known to play an important role in DSB repair by HR. In mammalian cells, RAD51C participates in branch migration and Holliday junction resolution (Liu et al., 2004). RAD51C mutation in cell lines results in spontaneous chromosomal aberrations and hypersensitivity to DNA damaging agents (Godthelp et al., 2002; Drexler et al., 2004). In addition, disruption of RAD51C always leads to embryonic lethality in mammals (Shu et al., 1999; Deans et al., 2000; Kuznetsov et al., 2007).

In contrast, RAD51C mutation in higher plants generally leads to a sterile phenotype, without affecting vegetative growth. Previous studies in *Arabidopsis* demonstrated that AtRAD51C is required for HR and SPO11-dependent DSB repair. The *Atrad51c-1* mutant shows chromosomal fragmentation, which can be suppressed in the *spo11-1 atrad51c-1* double mutant (Abe et al., 2005; Li et al., 2005). Moreover, AtRAD51C-deficient plants are also sensitive to DNA damaging factors (Bleuyard et al., 2005). In this study, considerable numbers of chromosome fragments were also detected from metaphase I in *Osrad51c*, suggesting that DSBs were formed but not properly repaired. As rice *CRC1* mutation inhibits DSB formation (Miao et al., 2013), we employed *crc1* instead of *spo11-1* to generate the *Osrad51c crc1* double mutant. The massive chromosomal fragmentation phenotype in *Osrad51c* was completely suppressed by the deletion of CRC1, indicating that OsRAD51C likely acts downstream of CRC1 and plays an important role in the process of DSB repair.

In mice, RAD51C foci could still be visualized at the pachytene stage, when they were clearly observed as one or two distinct foci associated with each paired chromosome. Interestingly, the staining pattern was similar to that of MLH1, which serves as a marker of chiasmata at the late pachytene/diplotene stage. In addition, in Mlh1-deficient mice, the RAD51C foci were markedly reduced (Liu et al., 2007). These results suggest that RAD51C foci may represent the sites of crossovers, and provide support for the role of RAD51C in the late stages of HR. In our study, OsRAD51C signals were first observed at leptotene as punctuate foci; these increased in number and reached a peak at zygotene. However, in contrast to the findings in mice, the signal had completely disappeared at pachytene. The differences in the distribution pattern of RAD51C between mammals and plants suggest that functional differentiation has taken place during evolution.

### Timing of the appearance of unrepaired chromosomal fragments

Several studies on mutants with chromosome fragmentation have shown that the fragments appear at different stage of meiosis. In the *Arabidopsis Atxrcc3* mutant, chromosomal fragments first appear at diplotene, while in the *atrad51* mutant they occur at diakinesis. Another study demonstrated that mutation in rice RAD51C led to the appearance of chromosomal fragments at pachytene (Bleuyard and White, 2004; Li et al., 2004; Puizina et al., 2004; Kou et al., 2012). However, in the present study, the chromosomal fragments were first observed at early metaphase I. A possible reason for this discrepancy is that the unrepaired fragment of chromatids were stuck together by sister chromatid cohesion. It is recognized that cohesion between sister chromatids depends on the multi-protein complex known as the cohesin complex. In meiosis, the cohesin along sister chromatid arms is dissolved during the metaphase I to anaphase I transition, while centromeric cohesion is maintained by shugoshin proteins until meiosis II (Xiong and Gerton, 2010). Generally, DSBs are generated during leptotene and are ultimately processed into crossovers and non-crossovers at zygotene. The sister chromatid arms are held together during this process by cohesin until they are gradually released at metaphase I. We therefore suggest that this is why we did not observe DNA fragments during early prophase I in the *Osrad51c* mutant. As the meiotic cell cycle proceeded, uneven chromosome condensation occurred, and chromosomes with irregular shape were found at diakinesis and early metaphase I. Following the degradation of cohesin on the chromosome arms, acentric chromosome fragments could be seen randomly distributed in the nucleus. Finally, we speculate that due to differences in chromosome size and cohesin distribution patterns, or even methods of chromosome preparation, chromosome fragments may seem to appear at different meiotic stages in different studies (Luo et al., 2014).

### Meiotic DSB repair requires a proper structural platform

The cohesin complexes not only function in mediating sister chromatid cohesion but have also been shown to be required for DSB repair (Birkenbihl and Subramani, 1992; Sjögren and Nasmyth, 2001; Atienza et al., 2005). REC8 is a meiosis-specific component of the cohesin complex, and is involved in homologous pairing and recombination and monopolar orientation (Klein et al., 1999; Brar et al., 2006; Shao et al., 2011). REC8 is also a master regulator of chromatin structure and is required for AE formation (Pasierbek et al., 2001; Xu et al., 2005). AEs play a central role in establishing the leptotene chromosome and provide a structural platform for subsequent meiotic events. PAIR2 and PAIR3, two AE-associated proteins in rice, are involved in homologous pairing and recruiting recombination factors onto chromosomes. Although the exact relationship of PAIR2 and PAIR3 with AE proteins is still unclear, we speculate that they may be constituents of a structure that associates with AE, based on previous studies (Nonomura et al., 2006; Wang et al., 2011).

In this study, we found that normal loading of OsRAD51C was partially disrupted in the *Osrec8* mutant. However, in *pair2* and *pair3* mutants, almost no signals were detectable during the zygotene stage, suggesting that PAIR2 and PAIR3 provide a structural platform for the recombination intermediates. Once the platform is destroyed, all other recombination proteins then fail to be loaded normally. Thus, we hypothesize that DSB processing requires a proper structural chromosome platform mainly composed of AE proteins. It is also possible that DSBs in *pair2* and *pair3* are repaired using sister chromatids as templates.

## Conflict of interest statement

The authors declare that the research was conducted in the absence of any commercial or financial relationships that could be construed as a potential conflict of interest.

## Supplementary material

Supplemental Table 1 **The list of primers used in this study**.
