# Supplementary material for: OsRAD51C is essential for double-strand break repair in rice meiosis
Source: Front Plant Sci. 2014 May 7;5:167. doi: 10.3389/fpls.2014.00167 (PMC4019848; doi:10.3389/fpls.2014.00167)
Supplement: Supplemental Table 1 — The list of primers used in this study. [file DataSheet1.DOCX]

| **Purpose** | **Name** | **Sequence (5' to 3')** | |
| --- | --- | --- | --- |
|  |  | **Forward** | **Reverse** |
| Map-based cloning | C1 | CTCCTAGGGACAGTTTTAG | GCTATAGAAGTTTTAGAGTC |
|  | C2 | CACAATGTTTTGCTTATCCG | AAGTTTTACAGGTGCCATCAC |
|  | C3 | GAATTAGGTTAGACCGAGAC | CTCTGTCTTAGGTCATTTCG |
|  | C4 | CATCGGACCATGCCAGTC | GGCCAGTAGATACGGTTG |
|  | C5 | TGTATTGCGATTTTGTGAGC | TCAGATGTACCAGGTGAAG |
|  | C6 | AGGCCTGTCAAGGTGGAG | CGGTGACGTCTTCTATTTG |
|  | C7 | GATGATGGTACTAGTGGTG | CTGACATCGTGCATTATCG |
|  | C8 | CAAAGCTTTCCGTCTTCAC | GAGGTTGAAATTCACGCTG |
|  | C9 | AAAGTAGGACAAACCATCAG | CTACAGGAAATCAATGAGAAC |
|  | C10 | CTACATATAGCATCTACCTG | AAGGTTCGGTCTAAAGTTCT |
|  | C11 | GGGACAGAGTGAGTATCGGA | GAAGCATAACGTCATCTTGG |
| RNAi | CR1 | AACTCGAGGCAGAAGGGTGCATCAGTG | TGAGATCTGGGGACTTGTCCAGGAATC |
| Antibody production | CA1 | AGGATCCATGGAGATCGCCGACCTCCC | ACTCGAGAACACTGCTGGTGCTAGGCC |

**Supplemental Table 1. The list of primers used in this study.**
